# Transcriptomic Remodeling Occurs During Cambium Activation and Xylem Cell Development in *Taxodium ascendens*

**DOI:** 10.3390/cimb46110708

**Published:** 2024-10-23

**Authors:** Kebing Du, Youming Xu, Ningning Wang, Liyuan Qin, Jieyun Tao

**Affiliations:** College of Horticulture and Forestry Science, Huazhong Agricultural University, Wuhan 430070, China; kebingdu@mail.hzau.edu.cn (K.D.); wnn0607@126.com (N.W.); qly123@webmail.hzau.edu.cn (L.Q.); taojieyun0910@163.com (J.T.)

**Keywords:** *Taxodium ascendens*, transcriptome, vascular cambium, wood formation

## Abstract

*Taxodium ascendens* has been extensively cultivated in the wetlands of the Yangtze River in south China and has significantly contributed to ecology and timber production. Until now, research on *T. ascendens* genomics has yet to be conducted due to its large and complex genome, which hinders the development of *T. ascendens* genomic resources. Combined with the microstructural changes during cambium cell differentiation across various growth periods, we investigate the transcriptome expression and regulatory mechanisms governing cambium activity in *T. ascendens*. Using RNA sequencing (RNA-Seq) technology, we identified the genes involved in the cambium development of cells at three stages (dormancy, reactivation, and activity). These genes encode the regulatory and control factors associated with the cambial activity, cell division, cell expansion, and biosynthesis of cell wall components. Blast comparison revealed that three genes (*TR_DN69961_c0_g1*, *TRINITY_DN17100_c1_g1*, *TRINITY_DN111727_c0_g1*) from the *MYB* and *NAC* families might regulate transcription during lignin formation in wood thickening. These results illustrate the dynamic changes in the transcriptional network during vascular cambium development. Additionally, they shed light on the genetic regulation mechanism of secondary growth in *T. ascendens* and guide further elucidation of the candidate genes involved in regulating cambium differentiation and wood formation.

## 1. Introduction

*Taxodium ascendens* Brongn is a tree species from the Taxodiaceae family. It is indigenous to southeastern America and was introduced to China in the early 20th century. The species possessed an enlarged base trunk with the characteristics of moisture resistance. Since then, it has been widely planted in the plains and lakes of the middle and lower reaches of the Yangtze River [1]. Genetic factors determine tree growth and wood production. Perennial trees, such as poplar and *Cunninghamia lanceolata*, have been the focus of the majority of genomic research, whereas research on *Taxodium ascendens* tree genomics remains limited. Cambial activities influence the diameter growth of trees and the formation of wood.

In woody plants of the northern temperate zone, vascular cambium cells cease dividing and differentiating when they become dormant during winter. In spring, favorable environmental conditions return, and they resume dividing and differentiating. This meristem state experiences a periodic activity, formed by seasonal changes of perennial trees throughout the year [1,2]. Therefore, studying the annual activity of vascular cambium is a fundamental means of comprehending forestry science, essential for forestry production. Research on the mechanism of tree cambium activity and xylem cell development during various annual growth phases regarding gene regulation is limited, but it is crucial to understanding tree growth and wood formation. This study utilized sectioning to acquire samples of particular developmental stages and the transcriptome from diverse phases of wood development. The genes that encode lignin and cellulose biosynthesis were discovered to be undergoing strict developmental stage-specific transcriptional regulation.

Previous research suggests that the differentiation of vascular cambium in plants is a complex process that involves multiple sequential events such as cell division, cell characterization, and cell differentiation. This process is subsequently modulated by a signal transduction network and is regulated by a complex gene network [3,4,5]. In recent years, significant attention has been given to transcriptome studies. Many regulatory factors and differential genes that contribute to cambium activity, cell division, cell expansion, and cell wall biosynthesis and modification have been identified [6,7]. Illumina sequencing was utilized to identify several miRNAs that exhibited significant expression changes within poplar. Additionally, 27 lignin-related genes were co-expressed in lignified tissues, associated with phytohormones and stress [8,9]. Other studies have conducted transcriptome sequencing and gene expression profiling in the cambial zone and differentiating the xylem of Japanese cedar, as well as the identification of stage-specific microRNAs in the cambium during poplar dormancy [10,11].

Much of the research on gene regulation of the vascular cambium cycle has focused on regulating plant hormones. For instance, auxin [12,13,14] and cytokinin (CTK) regulate the development of vascular cambium cells [15]. Ethylene and brassinosteroids (BR) also play significant roles in vascular development [16,17]. In addition, the regulation of the activity cycle of the vascular cambium necessitates certain transcription factors, including lonesome highway (LHW) and target of monopteros 5 (TMO5) from the bHLH transcription factor family. *TMO5/LHW* dimers encourage cell division by transducing signals to neighboring procambium cells [18]. Additionally, a poplar *HD-ZIP III* member, *PtrHB4*, plays a significant role in developing vascular bundles [19,20]. Some proteins within the R2R3-MYB family of transcription factors are involved in lignin biosynthesis in cell walls [21,22,23,24]. In Poplar, *PtrMYB2*, *PtrMYB3*, *PtrMYB20*, and *PtrMYB21* are significant in producing secondary cell walls [25]. Additionally, NAC transcription factors promote secondary wall biosynthesis in *Populus trichocarpa* [26,27]. *SND1*, *NST1*, *NST2*, *VND6*, and *VND7* have been discovered to enhance the production of the secondary wall by activating genes associated with MYB-like protein factors, including *MYB58*, *MYB63*, and *MYB46*. These factors cooperatively regulate lignin synthesis of in the secondary wall [24,28].

Lignin is a crucial element of the plant’s secondary cell wall (SCW). Physiological and genetic evidence support the biosynthesis of lignin, which results in stress resistance in plants during cellular processes [29,30]. Cell wall thickening occurs due to the deposition of lignin, cellulose, hemicellulose, and cell wall proteins [25,31], and it promotes wood formation, which involves programmed cell death. Various studies have examined the transcriptional regulation of wood formation [32,33,34].

With the rapid development of transcriptome sequencing technology, life science research has accelerated into the era of extensive data analysis. Bioinformatics offers the possibility of uncovering the code of life in the face of extensive genetic information in various species [35]. Transcriptome sequencing measures gene expression in specific cell states, comparing differences in transcriptome between individuals to explain differences at different developmental stages on a molecular level. Research on *T. ascendens* focuses primarily on physiology, ecology, and chemical composition. The research into the gene and transcriptome of *T. ascendens* is limited. Thus, this study examines *T. ascendens*, a significant commercially viable tree species. RNA extraction and sequencing were conducted on the vascular cambium of *T. ascendens* during various developmental stages (dormant, reactivating, and active). Furthermore, electron microscope sections were used to observe the ultrastructural changes of the vascular cambium. The study systematically examines cellular and genetic differences across various developmental stages of *T. ascendens*’ vascular cambium using microscopy and bioinformatics. It aims to gain insight into the differential gene enrichment of the transcriptome throughout the dormant, reactivating, and active stages while exploring the potential regulatory effects of transcription factors. In this way, we can gain a deeper understanding of the cambium transcriptome’s response to seasonal change and its regulation mechanisms, leading to insights into the genetic regulation of secondary growth in *T. ascendens*. We aim to identify the critical regulatory factors responsible for wood formation and provide a theoretical foundation for enhancing the quality of wood produced by *T. ascendens* plantations.

## 2. Material and Method

### 2.1. Plant Material

The cambium samples were acquired from the stems of *T. ascendens* (15 m tall, 20 cm in diameter at breast height) growing under natural conditions on the campus of Huazhong Agricultural University in Wuhan, China. (30.50′ N, 114.33′ E). Five trees of *T. ascendens* were chosen based on healthy growth and plant spacing. Stems were selected at the height of 1.3 m, rapidly frozen in liquid nitrogen, and stored at −80 °C in a refrigerator. The samples were collected in mid-December 2020 during dormancy (CS-1), in March 2021 during reactivation (CS-2), and in July 2021 during activity (CS-3). Six samples were taken during each period.

### 2.2. Anatomical Structure

The anatomical structure was observed through paraffin sectioning [36]. Producing paraffin sections involved several steps, including sampling, fixation, dehydration, infiltration, embedding, sectioning, adhering, staining, clearing, and sealing.

The cutting material had a thin and small size, less than 1.5 mm thickness, and an area of less than 5 × 5 mm^2^. The fixation period was 3–4 h. To dehydrate it, 30% ethanol was used, followed by 50%, 70%, 80%, 95%, and 100% for comprehensive dehydration. To fix it, 2.5% glutaraldehyde was utilized, and the volume of the fixation liquid was 5–10 times greater than the sample volume. Each dehydration step consumed 10–20 min, with a volume of liquid more than ten times that of the sample. The sample was first treated with chloroform to enhance permeability, followed by epoxy resin for 3–5 h to ensure complete penetration. The permeable sample was then placed in a mold, kept at 35 °C for 12 h, and left for 24 h. The thickness was 1 μm, and the cut section was flat and wrinkle-free when attached to a slide for analysis. For staining, the section was immersed in 1% solid green dyeing solution for 30–60 s, followed by dehydration in three cylinders of anhydrous ethanol. For transparent sealing, the sections were sliced into clean xylene for 5 min until transparent and sealed with neutral glue (Macklin, Shanghai Maclin Biochemical Technology Co., Ltd., Shanghai, China). Finally, microscopic examination and photography were conducted by Motic BA310T and Moticam 2506 (Macaudy Industrial Group Co., Ltd., Schertz, TX, USA).

### 2.3. Total RNA Extraction

Total RNA was extracted from the vascular cambium using TRIzol according to the manufacturer’s instructions for the Plant RNA Purification Reagent (Invitrogen, Carlsbad, CA, USA). The concentration and purity of the extracted RNA were measured using Nanodrop2000 (NanoDrop Technologies, Wilmington, DE, USA), while RNA integrity was determined through agarose gel electrophoresis. For a single library construction, 1 μg of total RNA with a concentration of ≥50 ng/μL and OD_260/280_ between 1.8–2.2 was required.

### 2.4. cDNA Library Preparation and Transcriptome Sequencing

Illumina sequencing was conducted at Shanghai Majorbio Bio-pharm Biotechnology Co., Ltd. (Shanghai, China), using the Illumina Novaseq 6000 platform. The RNA-seq transcriptome library was prepared following Illumina^®^ Stranded mRNA Prep, Ligation (San Diego, CA, USA) using 1 μg of total RNA. Shortly, messenger RNA was isolated according to the polyA selection method by oligo (dT) beads and then first fragmented by fragmentation buffer. Secondly double-stranded cDNA was synthesized using a SuperScript double-stranded cDNA synthesis kit (Invitrogen, Carlsbad, CA, USA) with random hexamer primers. Then, the synthesized cDNA was subjected to end-repair, phosphorylation, and adapter addition according to library construction protocols. Libraries were size selected for cDNA target fragments of 300 bp on 2% Low Range Ultra Agarose followed by PCR amplified using Phusion DNA polymerase (NEB) for 15 PCR cycles. After being quantified by Qubit 4.0, the sequencing library was performed using the Illumina HiSeq Xten/NovaSeq6000 machine [37].

### 2.5. Transcriptional Fragment Assembly and Differentially Expressed Gene Analysis

The raw paired end reads were trimmed and quality controlled by fastp [38] with default parameters. Then, clean data from the samples were used to perform de novo assembly with Trinity [39]. To increase the assembly quality, all the assembled sequences were filtered by CD-HIT [40] and TransRate [41] and assessed with BUSCO (Benchmarking Universal Single-Copy Orthologs). The assembled transcripts were searched against the NCBI protein nonredundant (NR), Clusters of Orthologous Groups of proteins (COG), and the Kyoto Encyclopedia of Genes and Genomes (KEGG) [42] databases using Diamond to identify the proteins that had the highest sequence similarity with the given transcripts to retrieve their function annotations, and a typical cut-off E-value of less than 1.0 × 10^−5^ was set [42]. The BLAST2GO program was used to obtain the GO annotations of unique assembled transcripts for describing biological processes, molecular functions, and cellular components [43].

To identify differentially expressed genes (DEGs) between two different samples, the expression level of each transcript was calculated according to the transcripts per million reads (TPM) method. RSEM [44] was used to quantify gene abundance [44]. Essentially, differential expression analysis was performed using the DESeq2 [45]. DEGs with |log2FC| ≥ 1 and FDR < 0.05 (DESeq2) were considered to be significantly differently expressed genes. In addition, functional-enrichment analysis including GO and KEGG were performed to identify which DEGs were significantly enriched in GO terms and metabolic pathways at Bonferroni-corrected *p*-value < 0.05 compared with the whole-transcriptome background. GO functional enrichment and KEGG pathway analysis were carried out by Goatools (v1.1.5) and Python SciPy software (v1.13.1), respectively.

### 2.6. qRT-PCR Validation and Expression Analysis

Real-time quantitative polymerase chain reaction (qRT-PCR) was performed as per protocol [46]: SYBR Green Master Mix 5 μL, ddH_2_O 3 μL, 0.5 μL forward primer, 0.5 μL reverse primer, and 1 μL cDNA were used. The amplification process included pre-denaturation for 30 s followed by denaturation for 10 s at 95 °C; annealing was performed at 60 °C for 30 s with 40 cycles. After the reaction, the melting curve was analyzed for 15 s at 60~95 °C to determine the specificity of the product. Each reaction was repeated three times. The melting curve analysis assessed primer specificity in real-time fluorescence quantification. A single peak in the melting curve indicated reasonable primer specificity. For this study, three groups of *Taxodium* at different growth stages, i.e., resting, reactivating, and active, were utilized. Three biological replicates represented each group, and each sample was analyzed in duplicate. The relative expression levels of the three growth stages were calculated using the 2^−ΔΔCt^ method.

### 2.7. Accession Number

The sequence reads of transcriptome sequencing have been deposited in the NCBI sequence read archive under accession number PRJNA1079915.

## 3. Results

### 3.1. High-Throughput Transcriptome Sequencing and Reads Assembly

Trinity was used to perform de novo assembly of all clean data samples, and the resulting assembly was optimized and evaluated (Table 1). The data showed that the number of assembled unigenes was 151,711 and the number of transcripts was 219,450, with an average N50 length of 1720 bp. Transcript refers to the assembled transcript, while unigene is the longest transcript within the transcript cluster. The average length of reads, percentage of Q30 (sequencing error rate less than 1%), and GC (guanine + cytosine) content were 983 base pairs, 93.64%, and 38.68%, respectively.

### 3.2. Principal Component Analysis of the Samples

**Figure 1:**
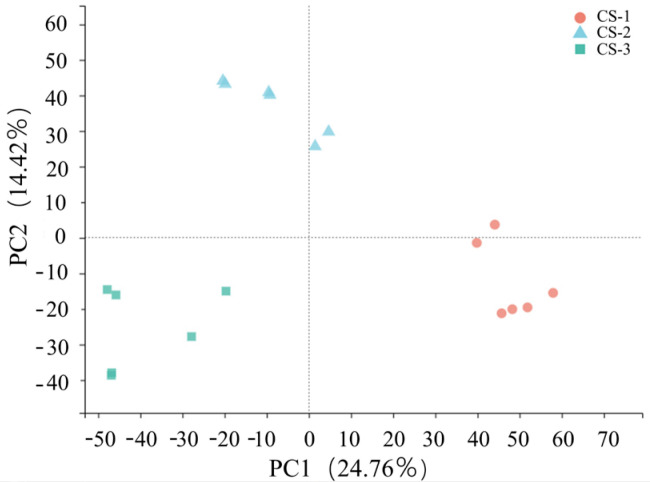
Principal component analysis (PCA) of the samples.

Principal component analysis (PCA) is a statistical technique that transforms a set of possibly correlated variables into a set of linearly independent variables called principal components through orthogonal transformation. The samples are then classified according to how similar their principal components are, which reflects the overall difference between each group of samples and the variability within each group. To centralize the formatting and perform logarithmic conversion of data, we utilized the MetaX4 software (v2.1.2). Since *T. ascendens* was sampled in its natural habitat, it was essential to establish the biological identity of the samples. Figure 1 shows the significant differences between samples taken during different periods, whereas those taken within the same period are comparable, providing reliable outcomes.

### 3.3. Differentially Expressed Genes Between Different Samples

**Figure 2:**
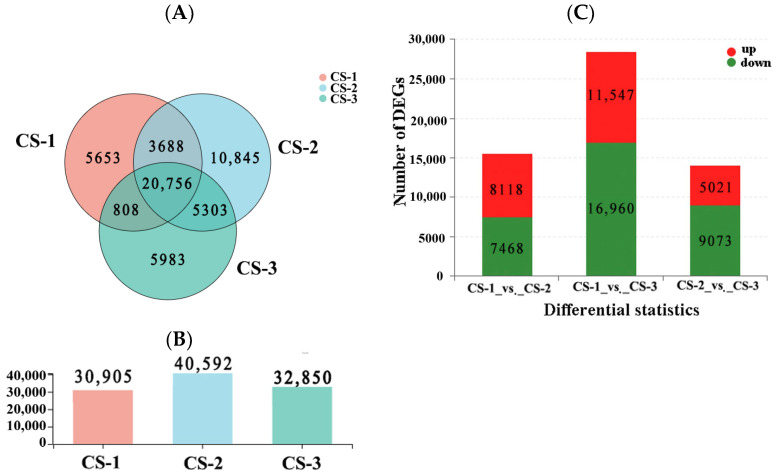
Venn diagram of the differentially expressed genes (DEGs) (**A**), the numbers of all genes within each period (**B**), and the up- and down-regulation bars of the differentially expressed genes (**C**). Note: For the CS-1 vs. CS-2 group, CS-1 is the control group and CS-2 is the experimental group, the same as below. Multiple of difference 2.0, FC ≥ 2.0 or FC ≤ 0.5.

A Venn diagram was utilized to study differentially expressed genes in the cambium across three stages of *T. ascendens*. It was determined that the three stages were co-expressed by 20,756 genes (Figure 2). Specifically, the experimental dormant stage (CS-1) consisted of 30,905 genes, the reactivating period (CS-2) consisted of 40,592 genes, and the active stage (CS-3) consisted of 32,850 genes. In addition, a comparison was made between the reactivating and dormant cambium libraries, resulting in the identification of 15,518 variant genes, with 8118 up-regulated and 7468 down-regulated. The transcriptional abundance of these critical switches displayed significant changes in the expression of differentially expressed genes at various stages of vascular cambium development.

### 3.4. Microscopic Changes of Vascular Cells in Different Developmental Stages

**Figure 3:**
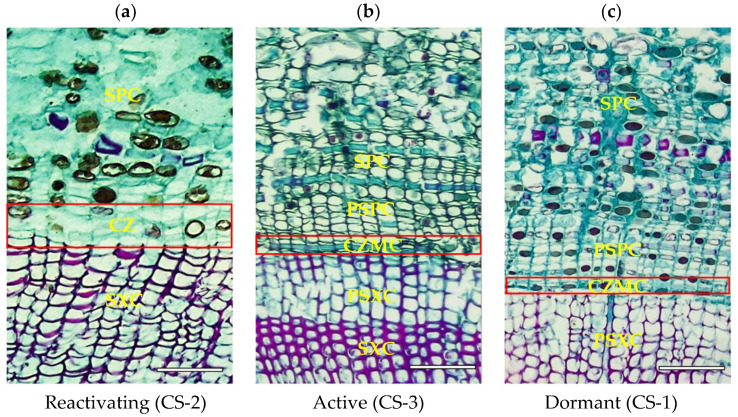
Cross section of vascular cambium of *T. ascendens* at different stages (Bar = 100 μm). Note: SPC-Secondary phloem cells of previous year; CZ-cambium zone (including 1–2 layers of cambium mother cells and newly differentiated secondary phloem cells); SXC-Secondary xylem cells of previous year; PSPC-Primary secondary phloem cells; PSXC-Primary secondary xylem cells; CZMC-Cambium zone mother cells.

The cross sections of the vascular cambium of *T. ascendens* at various developmental stages are depicted in Figure 3, which shows the section of the vascular cambium during the dormant period, when the cambium band is hardly visible, and the cells exhibit characteristics of the dormant phase. The cells have a flat shape, thickened radial walls, and an elevated cell content, while the xylem tracheids are lignified. At the reactivation stage, the thinning of cells along the cordwood wall was observed, while the cells increased in the radial direction, acquiring a spindle-like shape. The number of layers of cambium cells during this stage was between three and four, and the cambium started dividing. At the active stage, the number of layers of cambium cells reaches seven to eight, and cell volume gradually increases. At this time, cambium activity is at its peak. The cell wall is thinner and the staining is lighter, indicating that the cambium has reached its division peak.

### 3.5. Transcriptional Regulation Gene Expression Profile Analysis

**Figure 4:**
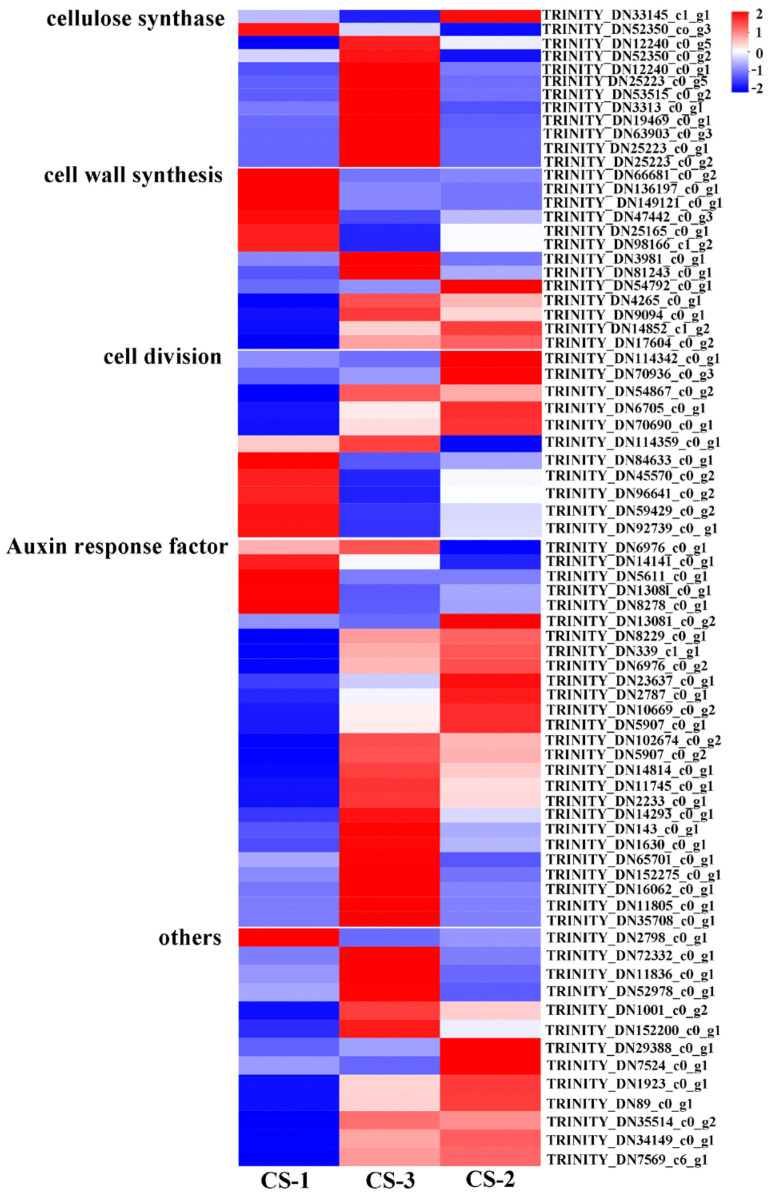
Heat map of dynamic changes of the genes involved in regulating cell division and differentiation in cambiums. Note: CS-1 dormant cambium; CS-2, reactivating cambium; CS-3, active cambium.

Genes were selected according to the annotation information in the NCBI-NR (NCBI non-redundant protein library) comprehensive database. Many genes regulate transitioning from dormant to reactivating and then to active, exhibiting significant differences and high dynamics (Figure 4). These genes include cellulose synthase (cellulose synthase-like protein D, cellulose synthase A catalytic subunit 4, and cellulose synthase catalytic subunit), cell wall synthesis-related genes (cell wall alpha-1,3-glucan synthase AGs1-like, cell wall synthesis protein Kre9-like, cell wall protein RBR3), cell division-related genes (cell division control protein 2 homolog A-like, putative cell division cycle ATPase, cell division control protein 3-like, cell division cycle 20.2, cofactor of APC complex like, and cell division control protein 48-like), Auxin response factor (auxin-induced like protein, auxin-responsive protein SAUR71, putative Auxin response factor 6/8, auxin-responsive protein IAA7), and so on. These genes were significantly overexpressed in the reactivating and active phases, consistent with the expectation of active cell division. Xylem development is a complex and orderly biological process, including the proliferation of vascular cambium cells, differentiation and expansion of xylem mother cells, cellulose and hemicellulose synthesis and lignin precipitation in the secondary cell wall, and programmed cell death, etc. In this study, most of the genes involved in xylem development were upregulated from the dormant period to the reactivating period, especially the genes involved in cellulose synthesis, cell wall synthesis, and cell division (Figure 4).

### 3.6. KEGG Pathway Enrichment Analysis of Differentially Expressed Genes

**Figure 5:**
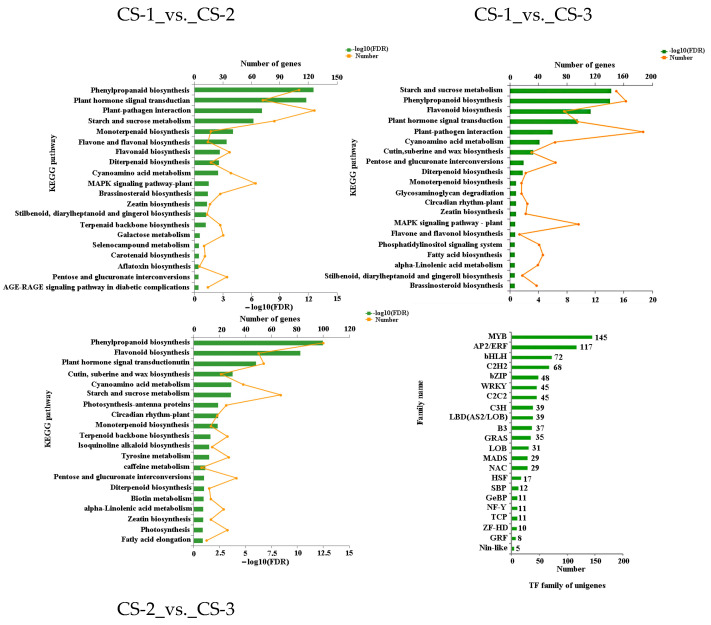
KEGG Pathway enrichment of differentially expressed genes in different periods.

During the reactivating period, phenylpropanoid biosynthesis was the most significantly enriched pathway, followed by the plant hormone transduction pathway (Figure 5). This suggests that the plant hormone response is active during the reactivating stage (CS-2) compared to the dormant period (CS-1). It is important to note that many genes participate in the brassinosteroid biosynthesis pathway. The starch and sucrose metabolism stood out significantly during the active period (CS-3) compared to the dormant period. Numerous genes react in the pathway of plant–pathogen interaction. From the reactivating to the active phase, the phenylpropanoid biosynthesis pathway was the most critical enrichment, followed by the flavonoid biosynthesis pathway. Compared to CS-1_vs._CS-2, the enriched genes were substantially reduced in CS-1_vs._CS-3 in the pathways of plant hormone transduction; flavonoid biosynthesis; brassinosteroid biosynthesis; and stilbenoid, diarylheptanoid, and gingerol biosynthesis. This means the activity of these pathways may be gradually diminishing from the reactivating stage to the active period.

### 3.7. Screening of MYB and NAC Transcription Family Genes Related to Wood Formation

In the *Taxodium* transcriptome, 888 transcription factors from 32 families were identified. Figure 5 shows the top 22 transcription factor families based on the number of members (Supplementary Table S1). The top five families were MYB, AP2/ERF, bHLH, C2H2, and BZIP. In addition, transcription factors exhibit significant differential expression patterns across different time stages, implying variations in transcriptional regulation mechanisms.

**Figure 6:**
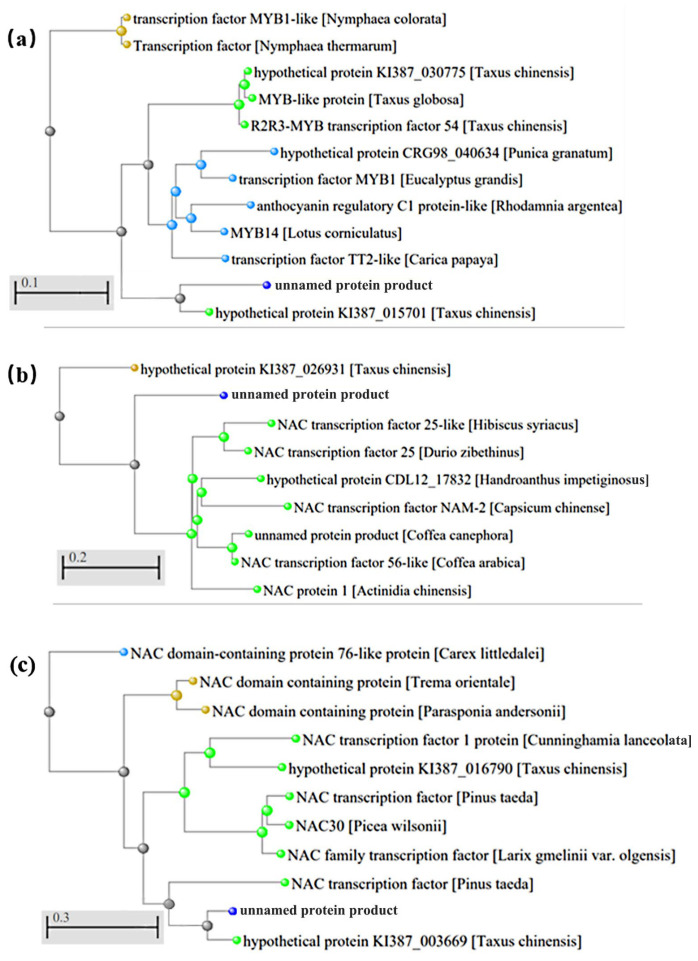
Phylogenetic tree of three genes in the *NAC* and *MYB* families. Note: (**a**): TRINITY_DN69961_c0_g1; (**b**): TRINITY_DN17100_c1_g1; (**c**): TRINITY_DN111727_c0_g1.

Previous studies have found that numerous genes of the MYB family are involved in the biosynthesis of lignin in cell walls (Supplementary Table S2). Specifically, *AtMYB58*, *AtMYB63*, and *AtMYB85* in the R2R3-MYB subgroup, as well as *AtMYB52*, *AtMYB54*, and *AtMYB69* in subgroup 21 [19,21,22,23] were identified in previous research as being linked to lignin biosynthesis. In this study, we selected MYB family genes to screen for those related to lignin biosynthesis and wood formation. A comparison was conducted between 145 genes of the MYB family in the *T. ascendens* and *Arabidopsis* genome, resulting in the comparison of a total of 20 genes. These 20 genes were annotated using the TAIR resource library (https://www.arabidopsis.org/)(accessed on 30 May 2022). In the *Arabidopsis* genome, *AT1G79180* belongs to the *R2R3-MYB* (2R) subfamily. This gene positively regulates secondary cell wall biosynthesis and participates in lignin biosynthesis. In *T. ascendens*, the gene *TRINITY_DN69961_c0_g1* was compared with *AT1G79180*, indicating a potential role in positively regulating the secondary cell wall and participating in lignin biosynthesis (Figure 6). The biosynthesis of the secondary cell wall and lignin is crucial for wood formation.

Like the MYB family, the NAC family comprises several genes involved in the biosynthesis of lignin in the cell wall. Blast comparison was performed on 29 genes of the NAC family between *T. ascendens* and the *Arabidopsis thaliana* genome, and 17 genes were differentially expressed (Supplementary Table S3). Meanwhile, seven genes were analyzed and annotated, revealing that two are associated with plant secondary cell wall biosynthesis. Among them, the *TRINITY_DN17100_c1_g1* gene and *AT4G36160* gene exhibit a 90.48% sequence similarity (Figure 6). *AT4G36160*, known as the transcription factor NAC076, is annotated as positively regulating secondary cell wall biosynthesis and the differentiation of xylem tracheid or conduit member cells. It encodes the NAC domain expressed during xylem development, and overexpression of this protein leads to abnormal secondary cell wall growth. Complementing certain cell wall defects in *SND1/NST1* double mutants, it is plausible to suggest that this gene in *Taxodium* positively regulates the biosynthesis of secondary cell walls and the differentiation of xylem tracheid cells. Additionally, the *TRINITY_DN111727_c0_g1* gene was found to identify another *Arabidopsis thaliana* gene, *AtNAC070* (*AT4G10350*), which has been described in the TAIR resource library as regulating the biosynthesis of secondary cell walls and the development of root caps. The similarity between this gene and *AtNAC070* is 88%, indicating that the gene may also be involved in regulating the biosynthesis of secondary cell walls in the vascular cambium of *Taxodium* (Figure 6).

The expression of the *TRINITY_DN17100_c1_g1* gene varied across developmental stages, as detailed in Supplementary Table S4. No significant difference was observed between the dormant and reactivating stages, with low expression levels. However, high expression levels were observed in the active stage, also associated with wood formation. The expression level during the active stage differed significantly from those during the dormant and reactivating stages. Combined with BLAST results, this gene is highly related to the regulation of wood formation. There was no significant difference in the expression levels of *TRINITY_DN111727_c0_g1* across the three periods. The protein sequences of the *TRINITY_DN69961_c0_g1* gene in *Taxodium* were BLASTP in the NCBI database, and homologous sequences were selected for constructing the phylogenetic tree. Fast minimum evolution parameters were employed for phylogenetic tree construction [47]. The results revealed that the gene is phylogenetically closest to the transcription factor MYB1-like (*Nymphaea colorata*) (Figure 6). The sequence with the highest identity was the hypothetical protein KI387_015701 (*Taxus chinensis*), and the percentage of identity was 88.76%. Regarding the NAC family gene *TRINITY_DN17100_c1_g1*, its closest phylogenetic relation was to the hypothetical protein KI387_026931 from *Taxus chinensis*, as shown in Figure 6. Another NAC family gene, *TRINITY_DN111727_c0_g1*, exhibits the nearest phylogenetic affinity to the NAC domain-containing 76-like protein from *Carex littledalei*. The maximum sequence identity is 84.2% with the hypothetical protein KI387_003669 from *Taxus chinensis*.

### 3.8. Verification of the Gene Expression Profiles by qRT-PCR

**Figure 7:**
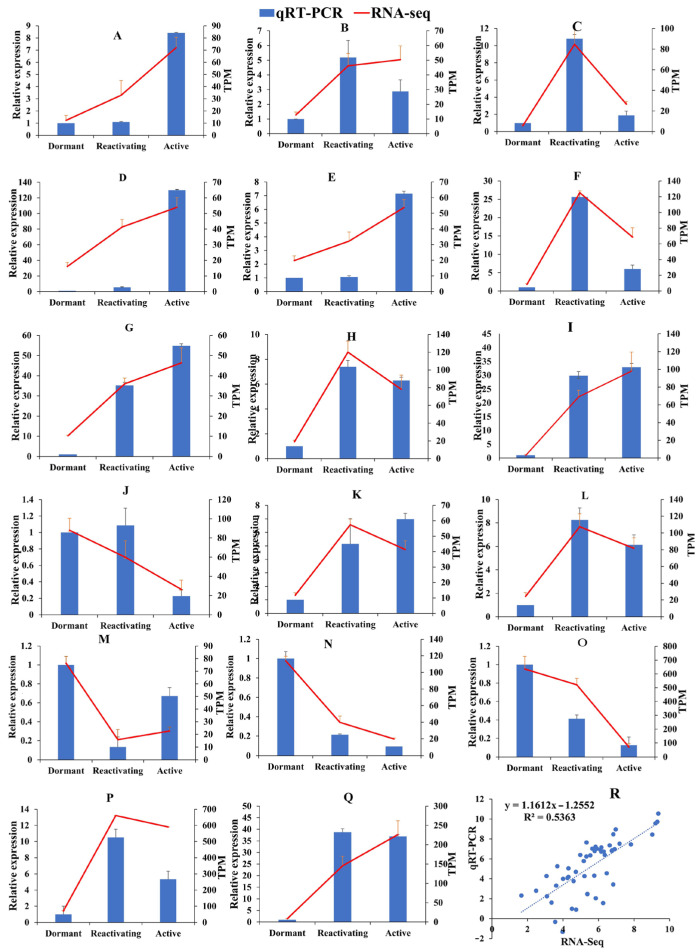
The expression consistency and correlation analysis between qRT-PCR and RNA-seq. Note: (**A**): *TRINITY_DN164_c1_g2*; (**B**): *TRINITY_DN2586_c0_g2*; (**C**): *TRINITY_DN594_c0_g1*; (**D**): *TRINITY_DN364_c0_g1*; (**E**): *TRINITY_DN12513_c0_g1*; (**F**): *TRINITY_DN30077_c0_g1*; (**G**): *TRINITY_DN3390_c0_g1*; (**H**): *TRINITY_DN17130_c0_g1*; (**I**): *TRINITY_DN5345_c0_g1*; (**J**): *TRINITY_DN8031_c0_g1*; (**K**): *TRINITY_DN89_c0_g1*; (**L**): *TRINITY_DN1923_c0_g1*; (**M**): *TRINITY_DN28161_c0_g1*; (**N**): *TRINITY_DN15316_c0_g1*; (**O**): *TRINITY_DN2798_c0_g1*; (**P**): *TRINITY_DN1049_c0_g1*; (**Q**): *TRINITY_DN6538_c0_g1*; (**R**): Correlation analysis of RNA-seq and qRT-PCR.

To validate the accuracy of RNA-Seq, we screened 17 differentially expressed genes and analyzed their expression through real-time quantitative PCR (qRT-PCR) (Figure 7). The sequences of these primers are listed in Supplementary Table S4. These genes encompass NAC, C3H, MYB, and GRAS transcription factor family genes, as well as the genes enriched in plant hormone metabolic transduction, such as auxin influx vector gene *TRINITY_DN5345_c0_g1*, auxin signal transduction gene *TRINITY_DN8031_c0_g1*, and cytokinin signal transduction gene *TRINITY_DN89_c0_g1* mediates. Other genes include those involved in brassinolactone signal transduction, gibberellin protein metabolism, and regulatory factors in the abscisic acid signal transduction pathway. The results showed that the expression patterns of each gene were consistent across both methods. The correlation coefficient between RNA-seq and qRT-PCR was 0.7323, with a reliability of 53.63%, indicating that the RNA-seq results are reliable.

## 4. Discussion

### 4.1. Gene Annotation and Enrichment Analysis

Wood is a significant natural resource in forests. Its formation is a secondary developmental process that is spatially and temporally regulated. It is crucial to comprehend the biological foundation of wood formation [48]. Gene coding regulates the activity of wood cambium, and transcriptome studies analyze gene expression and regulation at the RNA level to provide insight into the phenotype and function of organisms [49]. Some scholars suggest that transcriptional regulation may play an increasingly central role in regulating secondary growth [50]. In the present study, 18,407 unigenes were annotated and matched to the KEGG pathway database. KEGG gene enrichment analysis revealed that carbohydrate metabolism in the cambium was the most enriched pathway, followed by active signal transduction.

A total of 15,586 differentially expressed genes were identified during the reactivating and dormant stages of *T. ascendens*; 28,507 genes exhibited differential expression between the active and dormant stages, while 14,058 genes displayed differential expression between the active and reactivating stages. These results were remarkably different with *Populus tomentosa*. In this poplar species, a total of 8484 DEGs were found in the comparison between the dormant stage and the reactivating stage. The numbers of DEGs retrieved from the dormant stage vs. the active stage and the active stage vs. the reactivating stage were 6258 and 2690, respectively [51]. To comprehend the functions and biological pathways of these DEGs, we compared them with the KEGG and GO databases. It was determined that up-regulated genes of various secondary metabolisms were primarily associated with phenylpropanoid biosynthesis, flavonoid biosynthesis, and brassinosteroid biosynthesis. The transcriptome results reveal substantial phenylpropanoid synthesis during the reactivating stage, indicating the presence of active cell division activity. Meanwhile, in *Populus tomentosa*, the DEGs were associated with the various biological processes involved in cambium activity periodicity, such as the regulation of meristem growth, xylem development, cell division and expansion, cell wall biosynthesis, plant hormone signaling (such as auxin, gibberellin, abscisic acid), and wood formation [51].

### 4.2. Genes Involved in Cell Division and Secondary Wall Biosynthesis During Vascular Cambium Development

In plants, MYB transcription factors are typically divided into four categories based on the number of MYB domains present at the N-terminal. The R2R3-Myb subfamily is the largest category, which includes over 100 members in many species [52,53]. Numerous studies have demonstrated that *MYB* genes regulate various plant biological processes, including plant growth and development, cellular morphogenesis, primary and secondary metabolism, biological and abiotic stress response, and defense mechanisms [52]. Additionally, many genes are involved in lignin production in cell walls. In *A. thaliana*, R2R3-MYB proteins are classified into subgroups according to their phylogenetic relationships and functions. In poplar trees, *PtrMYB2*, *PtrMYB3*, *PtrMYB20*, and *PtrMYB21* all play vital roles in the secondary cell wall biosynthesis [25]. In this study, we hypothesize that the gene *TR_DN69961_c0_g1* in *T. ascendens* is a positive regulator of secondary cell wall biosynthesis and is involved in lignin biosynthesis, contributing to wood formation.

The NAC family is critical in regulating plant growth, development, and secondary wall growth, particularly in wood formation. Different levels of expression were observed in 17 out of the 29 NAC genes examined in this study. Previous research found that a specific group of NAC genes, *PtrWNDs*, can initiate the secondary wall biosynthesis process [54]. In *A. thaliana*, the overexpression of *PtrWND2B* and *PtrWND6B* resulted in increased expression of secondary wall biosynthesis genes. In this study, a comparison was conducted between 29 genes from the *NAC* family and the *A. thaliana* genome. The genes *TRINITY_DN17100_c1_g1* and *TRINITY_DN111727_c0_g1* were found to regulate the differentiation of secondary cell wall biosynthesis and xylem tracheid cells. Additionally, besides the *MYB* and *NAC* families, C3H-type zinc finger proteins were also discovered to play a role in secondary cell wall biosynthesis [28,55].

With the exception of the MYB and NAC families, the HD-Zip III transcription factor also plays critical roles in regulating cambium activity and xylem development. The three families have different action mechanisms and regulatory targets, forming a complex transcriptional regulatory network, which jointly promotes plant growth and development and helps the plant cope with environmental changes [19,20]. The plant-specific transcription factor HD-Zip III plays critical roles in the process of cell differentiation, such as embryogenesis, apical meristem formation, leaf polarity, and vascular differentiation. Especially in xylem development, HD-Zip III influences the differentiation of vascular cambium cells into secondary xylem by regulating the expression of the Class III HD-Zip gene. MYB family transcription factors mainly regulate fiber development through interaction with HD-Zip transcription factors. Unlike HD-Zip III and MYB, NAC family transcription factors are more involved in the regulation of stress response and developmental processes in plants.

## 5. Conclusions

The vascular cambium plays critical roles in wood formation as a result of the growth and differentiation of secondary phloem and secondary xylem. Our study provides the first description of the transcriptomic remodeling that occurs during cambium activation and xylem cell development in *Taxodium ascendens*. We identified the genes involved in the cambium development of cells at three stages (dormancy, reactivation, and activity). These genes encode the regulatory and control factors associated with cambial activity, cell division, cell expansion, and biosynthesis of cell wall components. Blast comparison revealed that three genes (*TR_DN69961_c0_g1*, *TRINITY_DN17100_c1_g1*, *TRINITY_DN111727_c0_g1*) from the *MYB* and *NAC* families might regulate transcription during lignin formation in wood thickening. These results illustrate the dynamic changes in the transcriptional network during vascular cambium development. 

## Figures and Tables

**Table 1:** Summary of *Taxodium ascendens* transcriptome.

Type	Unigene	Transcript
Total number	151,711	219,450
Total base	149,163,585	256,341,938
Largest length (bp)	25,463	25,463
Smallest length (bp)	201	201
Average length (bp)	983	1168
N50 length (bp)	1720	2087
E90N50 length (bp)	3679	2855
Fragment mapped percent (%)	61.28	79.12
GC percent (%)	38.68	38.77

Total number: sequence number of unigene/transcript assembled; Total base: the number of unigenes/transcripts assembled; N50: The assembled unigene/transcript is sorted from large to small according to the length, and the corresponding length of transcript is accumulated to half of the total length; E90N50: N50 of unigene/transcript with the top 90% expression; Fragment mapped reads: All sample clean reads were merged and compared with the assembled unigene/transcript, and the mapped rate was obtained.

## Data Availability

The original contributions presented in the study are included in the article/Supplementary Materials; further inquiries can be directed to the corresponding author.

## Supplementary Materials

The following supporting information can be downloaded at: https://www.mdpi.com/article/10.3390/cimb46110708/s1, Table S1: qRT-PCR primer sequences; Table S2: Functional summary of 21 transcription factor families in *Taxodium ascendens*; Table S3: Blast comparison results of MYB family between *Taxodium ascendens* and *Arabidopsis thaliana* genome; Table S4: 17 differential gene expressions of NAC family.
