# Supplementary material for: Transcriptomic Remodeling Occurs During Cambium Activation and Xylem Cell Development in Taxodium ascendens
Source: Curr Issues Mol Biol. 2024 Oct 23;46(11):708. doi: 10.3390/cimb46110708 (PMC11592639; doi:10.3390/cimb46110708)
Supplement: Supplementary file 1 [file cimb-46-00708-s001.zip › cimb-3222698-supplementary.pdf]

Supplementary Materials

Table S1. Functional summary of 21 transcription factor families in *Taxodium ascendens.*

Transcription factor	Number	Transcription factor	Number
MYB	145	LOB	31
AP2/ERF	117	MADS	29
bHLH	72	NAC	29
C2H2	68	HSF	17
BZIP	48	SBP	12
C2C2	45	GeBP	11
WRKY	45	TCP	11
LBD	39	NF-Y	11
C3H	39	ZF-HD	10
B3	37	GRF	8
GRAS	35		

Table S2. Blast comparison results of *MYB* family between *Taxodium ascendens* and *Arabidopsis thaliana* genome.

Gene_id	Overlapping gene id	Gene name	Score	Evalue	Identity (%)
TR_DN9370_c0_g1	AT1G57560	AtMYB50	43	0.00034	88.89
TR_DN26445_c0_g1	AT2G36890	AtMYB38	161	1.40×10^{-39}	80.8
TR_DN715_c0_g1	AT2G16720	AtMYB7	64	1.50×10^{-10}	79.25
TR_DN715_c0_g1	AT5G10280	AtMYB92	58	1.00×10^{-8}	100
TR-DN8039_c0_g3	AT3G46130	AtMYB48	73	2.60×10^{-13}	88.68
TR_DN85040_c0_g1	AT3G13540	AtMYB5	121	9.60×10^{-28}	82.95
TR_DN13205_c0_g1	AT4G05100	AtMYB74	104	1.20×10^{-22}	87.14
TR_DN5315_c0_g3	AT5G59780	AtMYB59	60	3.30×10^{-9}	100
TR_DN69961_c0_g1	AT1G79180	ATMYB63	67	2.90×10^{-11}	85.11
TR_DN16812_c0_g2	AT3G12720	AtMYB67	73	2.80×10^{-13}	97.44
TR_DN10333_c0_g1	AT3G47600	AtMYB94	64	1.90×10^{-10}	90
TR_DN6268_c0_g1	AT5G02320	AtMYB3R5	130	2.10×10^{-30}	84.04
TR_DN69988_c1_g1	AT1G66370	AtMYB113	77	2.70×10^{-14}	83.93
TR_DN2256_c0_g1	AT3G13530	AtM3KE1	167	1.10×10^{-41}	82.93
TR_DN7672_c0_g1	AT2G32080	AtPURA1	50	2.70×10^{-6}	100
TR_DN69961_c1_g2	AT5G62470	AtMYB96	79	5.50×10^{-15}	74.67
TR_DN11587_c0_g4	AT5G35550	AtMYB123	63	4.60×10^{-10}	94.29
TR_DN2685_c1_g1	AT5G15310	AtMYB16	63	2.60×10^{-10}	97.06
TR_DN164_c1_g1	AT5G18270	AtNAC087	50	2.60×10^{-6}	96.43
TR_DN15849_c0_g1	AT5G16560	AtKAN1	120	3.00×10^{-27}	82.95

Table S3. 17 differential gene expressions of NAC family.

Gene Id	PF ID	DNA domain	Family	E-value	CS2vsCS3	CS1vsCS2	CS1vsCS3
TR_DN20270_c0_g1	PF02365	NAM	NAC	1.4×10^{-19}	no|up	no|down	yes|down
TR_DN13667_c0_g2	PF02365	NAM	NAC	7.3×10^{-42}	yes|up	no|up	yes|up
TR_DN2234_c0_g2	PF02365	NAM	NAC	4.0×10^{-42}	no|up	yes|up	yes|up
TR_DN4750_c1_g2	PF02365	NAM	NAC	1.0×10^{-37}	no|up	yes|up	yes|up
TR_DN7925_c0_g1	PF02365	NAM	NAC	1.1×10^{-19}	no|down	yes|down	yes|down
TR_DN10034_c0_g1	PF02365	NAM	NAC	2.7×10^{-22}	yes|up	no|up	yes|up
TR_DN4284_c0_g1	PF02365	NAM	NAC	4.4×10^{-19}	no|up	yes|down	yes|down
TR_DN164_c1_g2	PF02365	NAM	NAC	1.1×10^{-40}	yes|up	no|up	yes|up
TR_DN6538_c0_g1	PF02365	NAM	NAC	9.3×10^{-24}	no|up	yes|up	yes|up
TR_DN44858_c0_g2	PF02365	NAM	NAC	1.8×10^{-21}	yes|up	no|up	yes|up
TR_DN16735_c0_g1	PF02365	NAM	NAC	3.1×10^{-31}	no|down	yes|up	no|up
TR_DN2150_c0_g1	PF02365	NAM	NAC	1.7×10^{-19}	yes|down	yes|down	yes|down
TR_DN18509_c0_g2	PF02365	NAM	NAC	6.5×10^{-42}	yes|up	yes|down	no|up
TR_DN17100_c1_g1	PF02365	NAM	NAC	7.0×10^{-40}	yes|up	no|down	yes|up
TR_DN17100_c0_g1	PF02365	NAM	NAC	1.1×10^{-39}	yes|up	no|up	yes|up
TR_DN2746_c0_g1	PF02365	NAM	NAC	1.1×10^{-38}	no|up	yes|down	yes|down
TR_DN1367_c0_g1	PF02365	NAM	NAC	1.2×10^{-23}	yes|up	no|up	yes|up

Note: no—not significant difference; up—up regulated; down—down regulated.

Table S4. qRT-PCR primer sequences.

Gene ID	Forward primer (5′-3′)	Reverse primer (5′-3′)
164	CTGGCTGGATACTGGAAAGCA	TACCTTGGCTGTCTTGGGAAG
2586	AGGAGAACAGTCCAAGAGGTG	TCAACATTCCTCAATGCAGCAA
594	CTTGGGTTCAGTTGGGCTAGA	ACTGCTGGTGAAGGCTGTTTA
364	GCACCATCTCTTGGCCCTAAC	ATTGACTGCTACGCACTCGTC
2048	AGAGCTGCTAAGTGACAACGAT	ATTCACCTGAAACGGTTGGAGT
12513	ATGTACAGCAGCAAGGACAGC	AGTGGACTGCCTCAGATGACA
30077	AACTGCGCAGGTTCTCCAG	GGATTGACACCGACGCCAT
3390	GAAGTGACGATTGCGGAGGC	GCTCACATCGCCGTTTCGAT
17130	CTGACAGCAACGAGGAGCTTG	CATCTCTGCACCAAGGACAGC
5345	CTGAGGAAGCAATTGTGGCG	AGAAGCACAGCTGAACCAGG
8031	AGGTTTCATCGTCCATGGAGAG	CCTCCACAACTTCAGTCCTCAT
89	GAATCTTGCCCAGTTGCTTCG	GCTGATAAGCCCTGTACTGCT
1923	CGGAACACAAGCTGGACGG	AGTCTTGCAGGAGGTCCAG
28161	GTCATGAGACTGGTTCGGCAT	AATCCACTGCGCTAATGAGCT
15316	TCCGCGGCGATTGACAATAAT	CTCGCTTCCCTTCATCTGCTG
2798	AAGGTGACAGAGAACAGAGGAT	AAGGTGACAGAGAACAGAGGAT
1049	GAGCTCTTCAGCACATCCTGG	AAGGATTAGCCATGGAAGGCC
6538	GACAGGAAAGATTCGTTGGCAT	GTGCCAAGATGGTATTGATGCA
